# LAMP: A Database Linking Antimicrobial Peptides

**DOI:** 10.1371/journal.pone.0066557

**Published:** 2013-06-18

**Authors:** Xiaowei Zhao, Hongyu Wu, Hairong Lu, Guodong Li, Qingshan Huang

**Affiliations:** 1 State Key Laboratory of Genetic Engineering, School of Life Sciences, Fudan University, Shanghai, China; 2 Shanghai High-Tech Bioengineering Co., Ltd, Shanghai, China; 3 Shanghai High-Tech United Bio-Technological R&D Co., Ltd, Shanghai, China; Uni. of South Florida, United States of America

## Abstract

The frequent emergence of drug-resistant bacteria has created an urgent demand for new antimicrobial agents. Traditional methods of novel antibiotic development are almost obsolete. Antimicrobial peptides (AMPs) are now regarded as a potential solution to revive the traditional methods of antibiotic development, although, until now, many AMPs have failed in clinical trials. A comprehensive database of AMPs with information about their antimicrobial activity and cytotoxicity will help promote the process of finding novel AMPs with improved antimicrobial activity and reduced cytotoxicity and eventually accelerate the speed of translating the discovery of new AMPs into clinical or preclinical trials. LAMP, a database linking AMPs, serves as a tool to aid the discovery and design of AMPs as new antimicrobial agents. The current version of LAMP has 5,547 entries, comprising 3,904 natural AMPs and 1,643 synthetic peptides. The database can be queried using either simply keywords or combinatorial conditions searches. Equipped with the detailed antimicrobial activity and cytotoxicity data, the cross-linking and top similar AMPs functions implemented in LAMP will help enhance our current understanding of AMPs and this may speed up the development of new AMPs for medical applications. LAMP is freely available at: http://biotechlab.fudan.edu.cn/database/lamp.

## Introduction

Resistance to antibacterial drugs is fast becoming a serious problem in all parts of the world. To address this problem, the Infectious Diseases Society of America launched the 10×’20 Initiative to develop 10 new antibacterial drugs by 2020 [1]. Antimicrobial peptides (AMPs) are indispensable components of innate defense mechanisms and make promising candidates for novel anti-infective agents. They are ubiquitous in nature and have been isolated from a wide variety of sources including bacteria, invertebrates, vertebrates and plants. AMPs are active against Gram-positive and Gram-negative bacteria, fungi, viruses and eukaryotic parasites when tested in the laboratory and in experimental animal systems [2]–[5]. Many candidate AMPs that offer benefits over existing drugs have been identified, but many have failed in clinical trials. However, there is little doubt that AMPs will enter the marketplace as valuable antimicrobial agents within the next 10 years [6]. To achieve this goal, the speed of translating newly discovered AMPs into clinical or preclinical trials will have to be accelerated. Recently, researchers have used a number of sophisticated approaches to develop AMPs. They include AMP mimetics, hybrid AMPs, AMP congeners, cyclotides and stabilized AMPs, AMP conjugates, and immobilized AMPs [7], [8].

An understanding of the role of the amino acid sequence on the specificity and activity of AMPs is essential to exploit them as antimicrobial agents. AMPs are involved in a variety of biological activity. Experiments have revealed that small changes in the primary structure of a peptide may lead to drastic changes in its specificity and activity. Previously, we designed and constructed of a novel AMP [9] and reported that a single residue alteration (K9L) rendered the peptide (KWKSFIKKLTSKFLHSAKKF) inactive. Sequence changes do not always render the peptide non-antimicrobial but can alter the minimum inhibitory concentration (MIC) of the AMP. Our studies [9] on the CP-P (KWKSFIKKLTSKFLHLAKKF) peptide and its analogs derived from the AMP of *cecropin A1, melittin* and *magainin*, showed that a single S16L mutation decreased the MIC of CP-P by almost a quarter. Several similar studies [10], [11] have strongly indicated that the primary structure of the peptide influences its antimicrobial activity.

A comprehensive database of AMPs with information about their activity and cytotoxicity is necessary for sequence-specificity and sequence-activity studies. Although several AMP related databases were developed, all these databases either cover only specific AMP families or contain a limited collection of AMPs. These shortcomings limit the accuracy and scope of comparative analysis tools such as BLAST and make it difficult for researches to find existing AMPs. Moreover, all currently available AMP databases exist separately and links between them are lacking. As Hammami and Fliss [12] pointed out, cross-links between these databases would make their use more efficient, and researchers would benefit from such synergy. With this in mind, we have created a functional database that aims to provide a full collection of AMPs with cross-linking between existing databases for researchers to facilitate development the AMPs as useful drugs.

LAMP, a database linking AMPs, is an integrated open-access database that was created to provide a useful resource and tools for AMP studies. LAMP is a manually curated database which currently holds 5,547 AMP sequences of which 3,904 are natural AMPs and 1,643 are synthetic peptides. LAMP was built based on our previous computer codes established for EnzyBase, and by combining antimicrobial peptide entries from all kinds of resources, including the existing database resources built by several other labs, especially the CAMP and the APD. There are 3,706 links to the CAMP and 1,972 links to the APD. The overall classification of AMPs in the LAMP is similar to that in the CAMP (3,201 experiment-validated, 863 predicted, and 1,491 patents). However, additional entries were also obtained from Swiss-Prot and literature, leading to more entries than the peptides in the CAMP. LAMP is freely available to academic and industrial scientists who are interested in exploring AMPs with the aim of improving their activity and deducing their cytotoxicity by mining and learning from the data in LAMP. To our knowledge, LAMP currently holds the most entries and is the first AMP database with cross-linking. Although LAMP processes more detail of each AMP, we still provide the comment function for each AMP. We believe our works would help researchers work on AMPs more efficiently and conveniently.

## Results and Discussion

### Database Description

LAMP was created as a useful resource for AMP studies. The AMPs in LAMP are short, less than 100 amino acid residues long, and include natural and synthetic AMPs. The AMPs in LAMP have been partitioned into three classes based on data source: experimental, predicted and patent.

**Figure 1:**
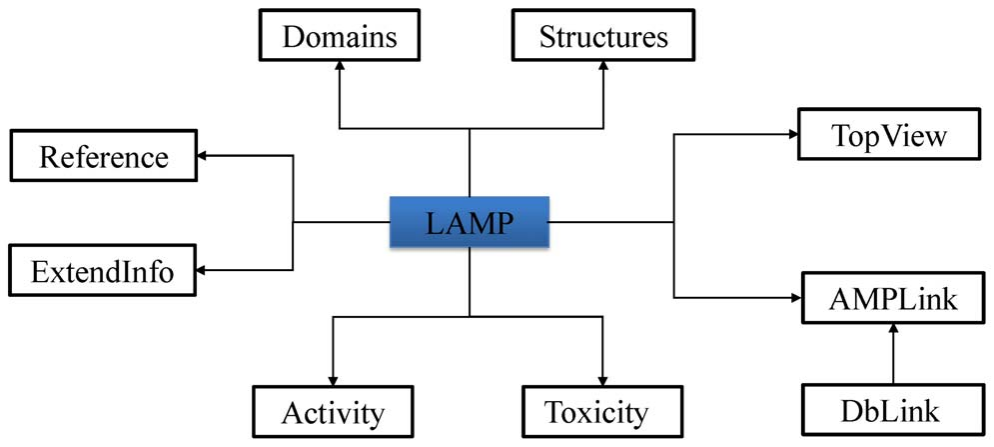
Schema of the LAMP database.

LAMP is composed of 10 relational tables in MySQL. Figure 1 show the schema of the database. Basic information related to sequence, protein definition, accession numbers, brief activity, taxonomy of the source organism is included in the LAMP schema. Domain and structure information of each AMP, if available, is stored in the Domains and Structures tables. TopView stores the top 10 AMPs that are most similar to each of the AMPs in LAMP. Cross-links to external databases like UniProt and other AMP database are recorded in AMPLink. Reference information related to each AMP is stored in Reference. Users can add comments to any of the AMPs and these comments are included in ExtendInfo. The antimicrobial activity data with MIC values and cytotoxicity data with Measurement of Hemolytic Activity (MHC) values for each AMP are stored in Activity and Toxicity respectively. DbLinks contains information from other AMP databases and their current status.

LAMP has a user-friendly web interface, so that users can easily query and retrieve information on AMPs. All the data in LAMP can be accessed and retrieved directly from the web browser. The database will be updated quarterly with additional sequences.

### Database Interfaces

**Figure 2:**
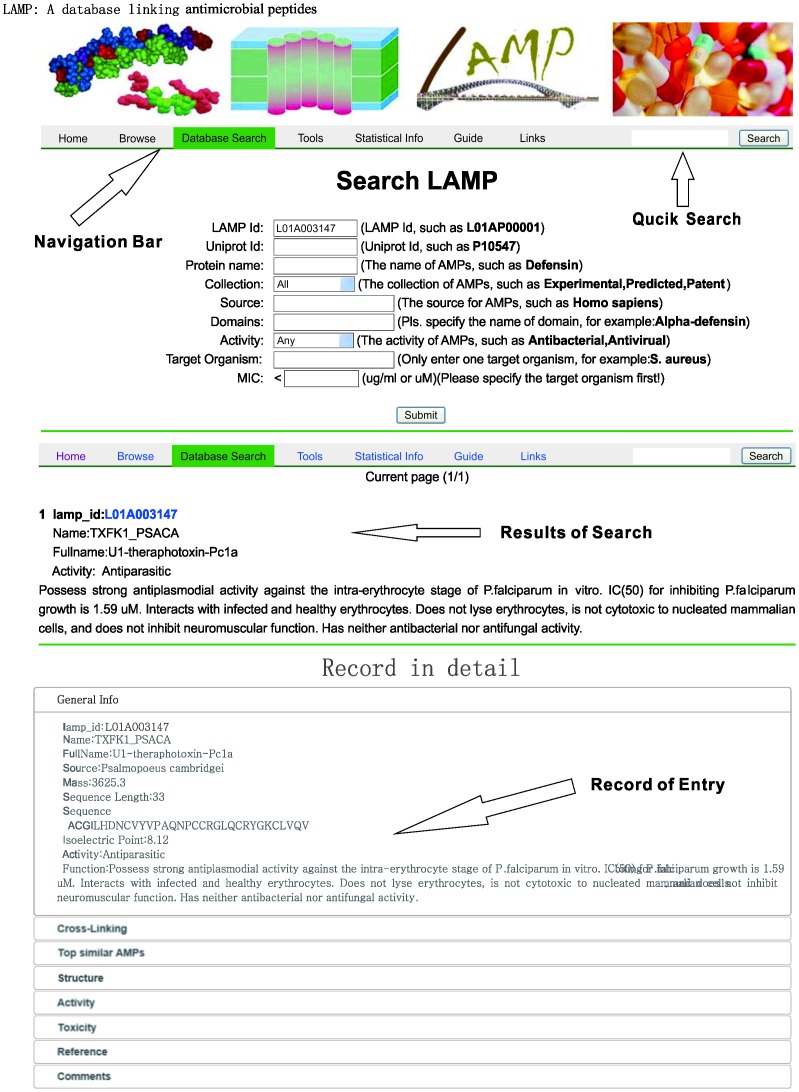
Screen shots of the LAMP search interface. The screen shots show the advanced search and result views. Please note that not all fields are shown.

A concise navigational interface that contains the database Browse, Search, Tools, Statistical information, Guide, and Links options was designed to generate a clearly structured database layout that enables fast and easy navigation (Figure 2).

**Figure 3:**
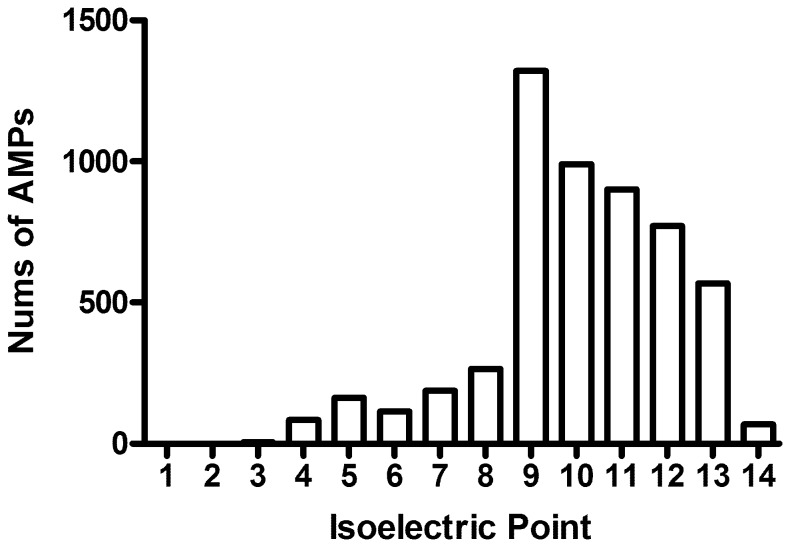
Distribution of calculated isoelectric points for the AMPs in LAMP. Every bar indicates the number of AMPs calculated to have their isoelectric point range from pI-1 to pI.

The Browse interface allows users to navigate not only the entire database but also the grouped AMPs by different views such as origin, data source and activity. In addition, the LAMP Browse interface contains a link that allows the download of all the AMPs in FASTA format. The Search interface can be used to retrieve specific information using either the quick or advanced options. A quick search can be performed using only keywords, while in the advanced search up to nine separate fields can be specified: namely, LAMP ID, UniProtKB ID, protein name, collection, source, domains, activity, target organism, and MIC value. The user can query the database by either one condition (excluding MIC, which also requires the type of target organism to be stated) or a combination of various conditions. For each AMP there is a results page with eight sections: general information, cross-linking, top similar AMPs, structures, antibacterial activity, toxicity, references and comments (Figure 3). General information consists of LAMP ID, protein name, protein full name, producer organism or source, protein mass, sequence length, sequence, calculated isoelectric point (pI), antibacterial activity, and simple functional annotation. Cross-linking provides hyperlinks to other public databases, such as UniProt, InterPro, PDB, and other AMP databases, which allows additional information on the AMP to be easily obtained. Top similar AMPs function provides the top similar AMPs produced by the BLASTP program. Equipped with the detailed antimicrobial activity and cytotoxicity data, the cross-linking and top similar AMPs functions will serve the study of sequence-activity better. The Tools interface permits BLASTP searches against LAMP to be performed. This allows users to input a peptide sequence and search the database for homologous sequences. The results can be copied and used for subsequent research. Because of limitations in the available disk space on the host site, a local BLASTP against NCBI databases has not been implemented; instead, a hyperlink to BLASTP on the NCBI website has been provided. The Statistical info interface provides data on the sources, domains and activity of the AMPs, and on the distribution of sequence length, protein mass, and calculated pI(see ‘Statistical description and findings’ section below for more information). The Guide interface provides simple instructions for potential users on how to use the functions of LAMP. The Links interface lists other AMP databases and their current status.

### Statistical Description and Findings

The current version of LAMP contains 5,547 AMPs, of which 5,362 AMPs have antibacterial activity, 1,161 AMPs have antiviral activity, 1,579 AMPs have antifungal activity, 14 AMPs have antiparasitic activity and 138 AMPs have antitumor activity. The AMP sequences range from 4 to 99 amino acids in length. The top 10 sources of the natural AMPs in LAMP are listed in Table 1. The majority of AMPs in LAMP (83.7%) have a calculated pI ranging from 9 to 13 (Figure 3).

**Table 1:** Top 10 sources of the natural AMPs in LAMP.

Rank	Producer organisms	Numbers of AMPs
1	***Mus musculus***	135
2	***Homo sapiens***	119
3	***Bos taurus***	86
4	***Rattus norvegicus***	62
5	***Apis cerana***	48
6	***Viola odorata***	43
7	***Macaca mulatta***	40
8	***Bombina maxima***	40
9	***Gallus gallus***	38
10	***Odorrana grahami***	36

The AMPs in LAMP contain a total of 230 domains; only 189 AMPs have a known 3D structure. The top 10 most abundant domains in LAMP are presented in Table 2. Knot1 is found in 171 of the AMPs and is the topmost domain. Four of the top 10 domains are subtypes of the Defensin domain and together they are found in approximately 11% of all the AMPs. Thus, it appears that many of the recorded AMPs are Defensin like. The top 10 AMPs for antimicrobial activity in LAMP are listed in Table 3.

**Table 2:** Top 10 domains in LAMP.

Rank	Interpro Id	Domain Name	Numbers of AMPs
1	IPR003614	Knot1	171
2	IPR007921	Defensin_beta/neutrophil	155
3	IPR017853	Defensin_beta-typ	143
4	IPR002053	Defensin_invertebrate/fungal	128
5	IPR013781	Alpha-defensin	113
6	IPR002901	Defensin_propep	112
7	IPR018392	Gamma-thionin	94
8	IPR013667	Mammalian_fefensins	93
9	IPR002482	Cecropin	89
10	IPR003646	Antimicrobial_frog_2	86

**Table 3:** Top 10 AMPs for antimicrobial activity in LAMP.

Rank	LAMP Id	Bacteria	MIC (μg/mL)
1	L01A003297	*S. newport*	0.01
2	L01A000547	*E. salicis*	0.02
3	L01A000547	*R. meliloti*	0.02
4	L01A000548	*E. salicis*	0.02
5	L01A000548	*R. meliloti*	0.02
6	L01A000549	*E. salicis*	0.02
7	L01A003100	*M. lysodeikticus*	0.02
8	L01A003197	*E. coli*	0.02
9	L01A000258	*N. crassa*	0.04
10	L01A003297	*E. coli*	0.04

### Comparison between LAMP and Other Databases

Over the past decade, a number of AMP-related databases had been developed http://biotechlab.fudan.edu.cn/database/lamp/links.php. DAMPD [13], AMSDb [14], APD [15], [16], CAMP [17] all cover AMP sequences from diverse origins. Other databases are more specialized and have focused to AMPs produced only by bacteria (BACTIBASE [18]), plants (PhytAMP [19]), shrimp (PenBase [20]), synthetic method (SAPD [21], [22]) and recombinant methods (RAPD [21]). Some databases have focused on specific families of AMPs, such as the defensins (Defensins knowledgebase [23], DADP [24]), cyclotides (CyBase [25]), enzybiotics (EnzyBase [26]) and peptaibols (Peptaibol [27]). In addition, AMPer [28] and BAGEL [29] serve as useful discovery tools for AMPs. Table S1 provides a brief comparison of LAMP and currently available AMP databases.

Compare to CAMP and APD databases, significant improvements available in LAMP include not only significantly more AMPs than CAMP and APD databases (5,547 in LAMP versus ∼ 3,782 in CAMP and 1,228 in APD), but also the unique Cross-links and Topview functions – this not available in CAMP or any other online resource. Currently LAMP (March, 2013) holds 5,547 AMPs, containing 3,904 natural and 1,643 synthetic AMPs by origin, containing 3,203 experimental, 1,491 patent and 853 predicted AMPs which may later be found not to be AMPs by data source. Also, LAMP possesses the particular MIC values and cytotoxic info for the study of the relationship between sequence and activity and future function development. Of the 3,051 natural peptides (excluding 853 predicted AMPs), there are 955 AMPs with MIC value and 178 AMPs with cytotoxicity data.

### Limitations and Future Prospects

As we all known, activity and toxicity are equally important for medical drugs. In the current LAMP, the cytotoxicity information of the AMPs is rare. In future, we will focus on collecting the cytotoxicity information and integrate the therapeutic index (MIC/MHC ratio) into LAMP so that the AMPs can be evaluated more accurately. Moreover, we plan to implement updates, continuously assess the data quality, and integrate some structural analysis tools and certain Web2.0 functions, like Wiki, into LAMP to improve its user interactivity and progress research in the field of AMPs design and structure function exploration.

### Conclusions

LAMP is a comprehensive and web accessible database of AMPs. The current version of LAMP has 5,547 entries (till March, 2013), including 3,203 experimental-validated, 1,491 patent and 853 predicted AMPs. The database can be queried either by simply using keywords or by combinatorial conditions searches. The tools and statistical information in LAMP will not only aid in enhancing our current understanding of AMPs and their mechanisms of action but may have implications in the development of new drugs for medical applications. LAMP now is available at http://biotechlab.fudan.edu.cn/database/lamp/.

## Materials and Methods

### Data Collection and Organization

All the AMP sequences were collected manually from the scientific literature or from the annotated UniProt and other AMP-related database. To ensure data quality, we sourced the information only from authoritative public databases and published scientific literature, as well as from patents. The AMPs collected in LAMP include natural AMPs and synthetic AMPs. Additional physicochemical data on the AMPs was either calculated via Bioperl programs or identified from the scientific literature. All of the collected information was classified and filled into 10 relational tables in MySQL. For each AMP, a unique identification number (i.e., LAMP ID) beginning with the prefix L was assigned. Each entry also contains general data, such as protein name, protein full name, producer organism, simple functional annotation and protein sequence, domain information, 3D structure, and relevant references. For the AMPs that already exist in the UniProt, InterPro [30], PDB [31] databases and/or other public AMP-related databases, hyperlinks to these databases were created in LAMP. Additional physicochemical data, including the calculated pI, and charge at the pI, are also provided. Moreover, MICs and MHC values are included, when the data are available.

### Web Interface and Application

LAMP was built on an Apache HTTP Server (V2.2.14) with PHP (V5.2.13) and a MySQL Server (V5.1.40) as the back-end. HyperText Markup Language (HTML), JQuery (V1.7.2) and Cascading Style Sheets (CSS) were used at the front-end. Apache, MySQL, and PHP were preferred because they are open-source software and platform independent, respectively, making them suitable for academic use. To perform the online sequence alignments, the BLASTP program (BLASTP V2.2.25+) was used for sequence homology searches against LAMP. The web server and all parts of the database are hosted at the Information Office of Fudan University, Shanghai, China.

## Supporting Information

Table S1 Comparison of LAMP with other available AMP databases.
(DOCX)
