# Supplementary material for: LAMP: A Database Linking Antimicrobial Peptides
Source: PLoS One. 2013 Jun 18;8(6):e66557. doi: 10.1371/journal.pone.0066557 (PMC3688957; doi:10.1371/journal.pone.0066557)
Supplement: Table S1 — Comparison of LAMP with other available AMP databases. (DOCX) [file pone.0066557.s001.docx]

Supplementary Material

Table S1. Comparison of LAMP with other available AMP databases

| **AMP Database** | **AMP origin** | **Search keywords** | **Data source** | **Number of entries** | **Last updated** | **MIC data** | **Cytotoxic data** | **Cross linking** |
| --- | --- | --- | --- | --- | --- | --- | --- | --- |
| LAMP | Natural & Synthetical | Antimicrobial;Antifungal;Anticancer;Antiparasitic | AMP Databases;UniProt;Literature; | 5547 | 2013 | Present | Present | Present |
| EnzyBase | Natural Enzybiotics | Lysin;Lysozyme;Bcteriocin | UniProt;Literature | 1144 | 2012 | Present | Absent | Absent |
| DADP | Amphibian defense peptides | Amphibian defense peptide;Antimicrobial;Antibiotic and taxonomy:amphibia | UniProt | 2571 | 2012 | Present | Present | Absent |
| DAMPD | Natural | Antimicrobial;Antifungal;Anticancer;Antiparasitic | UniProt | 1232 | 2011 | Absent | Absent | Absent |
| CAMP | Natural & Synthetical | Antimicrobial;Antifungal;Anticancer;Antiparasitic | GenBank;UniProt | 3782 | 2010 | Present | Absent | Absent |
| RAPD | Recombinantly-produced | Unknown | Literature | 179 | 2010 | Absent | Absent | Absent |
| APD | Natural | Antimicrobial;Antifungal;Anticancer;Antiparasitic | GenBank;UniProt | 1228 | 2009 | Present | Absent | Absent |
| BACTIBASE | Bacterial | Unknown | UniProt | 177 | 2009 | Present | Absent | Absent |
| PHYTAMP | Plant | Unknown | UniProt;Literature | 273 | 2009 | Present | Absent | Absent |
| PENBASE | Shrimp | Unknown | Est project on shrimp species | 28 | 2008 | Absent | Absent | Absent |
| DEFENSIN | Defensins | Defensin | UniProt;GenBank | 363 | 2008 | Present | Absent | Absent |
| PEPTAIBOL | Natural Peptaibols | Unknown | Literature | 317 | 2004 | Absent | Absent | Absent |
| AMSDB | Eukaryotic | Unknown | SwissProt;Literature | 895 | 2003 | Present | Absent | Absent |
